# Field Measurement-Based System Identification and Dynamic Response Prediction of a Unique MIT Building

**DOI:** 10.3390/s16071016

**Published:** 2016-07-01

**Authors:** Young-Jin Cha, Peter Trocha, Oral Büyüköztürk

**Affiliations:** 1 Department of Civil Engineering, University of Manitoba, Winnipeg, MB R3T 5V6, Canada; 2 GEI Consultants Inc., Woburn, MA 01801, USA; ptrocha@geiconsultants.com; 3 Department of Civil and Environmental Engineering, Massachusetts Institute of Technology, Cambridge, MA 02139, USA; obuyuk@mit.edu

**Keywords:** spectral analysis, system identification, sensor network, dynamic response, ambient vibration

## Abstract

Tall buildings are ubiquitous in major cities and house the homes and workplaces of many individuals. However, relatively few studies have been carried out to study the dynamic characteristics of tall buildings based on field measurements. In this paper, the dynamic behavior of the Green Building, a unique 21-story tall structure located on the campus of the Massachusetts Institute of Technology (MIT, Cambridge, MA, USA), was characterized and modeled as a simplified lumped-mass beam model (SLMM), using data from a network of accelerometers. The accelerometer network was used to record structural responses due to ambient vibrations, blast loading, and the October 16th 2012 earthquake near Hollis Center (ME, USA). Spectral and signal coherence analysis of the collected data was used to identify natural frequencies, modes, foundation rocking behavior, and structural asymmetries. A relation between foundation rocking and structural natural frequencies was also found. Natural frequencies and structural acceleration from the field measurements were compared with those predicted by the SLMM which was updated by inverse solving based on advanced multiobjective optimization methods using the measured structural responses and found to have good agreement.

## 1. Introduction

With improved materials and structural engineering techniques, tall buildings are increasingly in vogue in major cities, and these structures frequently must grapple with unique hazards, ranging from wind and seismic loadings, to reclaimed or unique foundation materials. Simultaneously, aging tall building infrastructure must be regularly monitored and inspected to ensure adequate safety. These challenges require an enhanced and particular understanding of the dynamic characteristics of individual structures. Such an understanding can be gained through instrumentation of tall structures and analysis of the resulting data to identify base-line dynamic properties and characterize structure responses to both ambient and high-energy excitations [1,2,3,4,5,6,7]. Through closer study of this dynamic behavior, advances may be possible in structural health monitoring and structural modeling of tall buildings, which can be applied in the design and lifecycle maintenance of the next generation of structures.

Although considerable research has been published on structural health monitoring, relatively less information is found on structural health monitoring methodologies, and measurement based damage detection of tall buildings. Nöldgen et al. [8] carried out studies of vulnerability and robustness of a security skyscraper subjected to aircraft impact. Irfanoglu [9] carried out extensive numerical aircraft crash simulations and thermal behavior analyses of the World Trade Center Tower. However, as discussed above, limited studies have been carried out to develop structural health monitoring methodologies and most of the research has been carried out using numerical simulation and synthetic data. Limited studies have also been reported on the identification of tall building dynamic characteristics based on field measurements. Brownjohn et al. [10] developed a lumped-mass model (LMM) based on finite element analysis (FEA) of the 66 story Republic Plaza building in Singapore to investigate dynamic characteristics of the building. The height of the building is 280 m, and the tower has a frame-tube structural system with an internal core wall connected to a ring of external columns by a horizontal steel framing system at every floor. Six different finite element models (FEM) of the building with varying level of complexity were developed using SAP 2000. The natural frequencies of the detailed structural model showed good agreement with natural frequencies determined from field measurements.

Kohler et al. [11] carried out dynamic property measurements of the moment-resisting steel-frame Factor Building at the University of California, Los Angeles (UCLA), to assess how forces are distributed over the building. This 17-story building had been monitored using 72 accelerometers from 2002 to 2003. From this study, the fundamental frequencies measured from earthquake data are about 0.5 to 0.1 Hz lower than those corresponding to ambient vibration recordings which indicate softening of the soil-building system with increased vibration amplitudes. Those frequencies reverted to pre-earthquake levels within 5 min of the earthquake. Skolnik et al. [12] carried out model updating and response prediction using a detailed finite element (FE) model and a lumped mass stick FE model based on measurement data for the Factor Building at UCLA. Nayeri et al. [13] implemented two system identification methods to the Factor building based on ambient vibration measurements. The first method is a natural excitation technique [14], and the second method is a time-domain identification technique for chain-like MDOF systems introduced by Masri et al. [15]. Lu et al. [16] developed a 2-dimensional (2-D) nonlinear structural model for seismic analysis of super tall buildings using Shanghai Tower. The 2-D model includes nonlinear beam-column elements and shear spring elements. Foti et al. [17] carried out output-only identification techniques and FE model updating on the Edifice A building, heavily damaged by the 2009 L’ Aquila earthquake.

Erol [18] used digital inclination sensors to monitor a tall building and a geodetic monument in Istanbul. The structures were separately monitored using bi-axes micro-radian inclinometers and GPS. The measured signals were analyzed using the Least Squares Spectral Analysis Technique (LSSA) to monitor deformation. Shi et al. [19] applied three output-only modal identification techniques to the ambient and forced vibration measurements of Shanghai World Financial Center. The methods were peak-picking method, random decrement based method, and Hilbert-Huang transform method. Yahyai and Amiri [20] proposed a procedure for ambient vibration monitoring method of tall telecommunication tower by analyzing the field measurement of Milad Tower. Rahgozar et al. [21] developed a simple mathematical model that is used to determine the optimum location of a belt truss reinforcing system on tall buildings such that the displacement due to lateral loadings such as wind and earthquake. Xia et al. [22] investigated the strain/stress development of the Guangzhou New Television Tower through the integration of finite element analysis and field monitoring during the construction state. Kang et al. [23] proposed a performance evaluation method for a tuned mass damper under typhoon based on the system identification and inverse wind load estimation for tall building. Celebi et al. [24] determined natural frequencies of the Green building, a 20-story tall structure located on the campus of the Massachusetts Institute of Technology, using field measurements from 36 accelerometers. Yi et al. [25] carried out dynamic characteristics studies using field measurements from a 420 m high tall building in Hong Kong during the passage of typhoons using accelerometers and global positioning system (GPS) mounted in the building.

In this paper, an existing array of 36 accelerometers in MIT’s Green Building is used to characterize the dynamic properties of the structure and validate a unique method for simplified, 3-D dynamic model development. Spectral and coherence analysis of field measurements from the accelerometer array are used to identify the Green Building’s natural frequencies, modes, and characteristic dynamic behavior, including base rocking and torsion. A condensed lumped mass model for 3-D dynamic analysis of the Green Building is developed using detailed finite element modeling, static condensation, and geometric transformations. Using measured acceleration responses, the developed model was updated by advanced multiobjective optimization method to match modal properties between the measurements and predicted from the numerical model. This model was used to perform modal and earthquake response studies of the Green Building. Comparison of model results with sensor measurements show good agreement. The model and modeling method developed in this paper will be used in future studies to investigate structural health, dynamic performance, and seismic response.

## 2. Measurement Based System Identification of Green Building on MIT Campus

**Figure 1:**
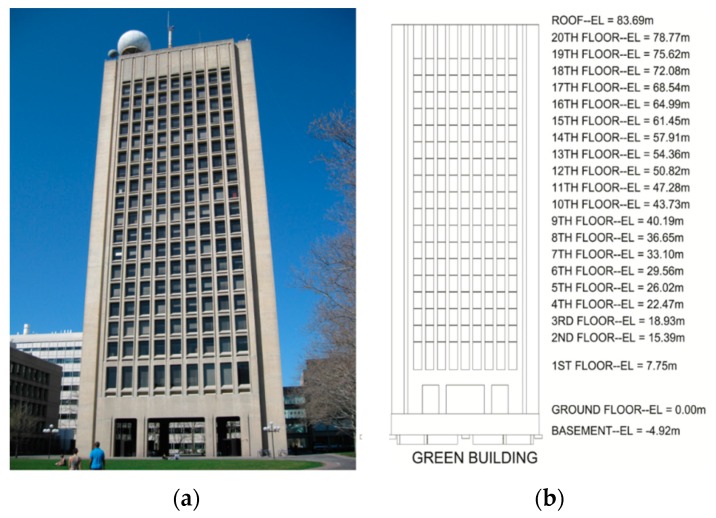
(**a**) Photograph of the Green Building’s south façade; (**b**) Elevation view of the south façade.

**Figure 2:**
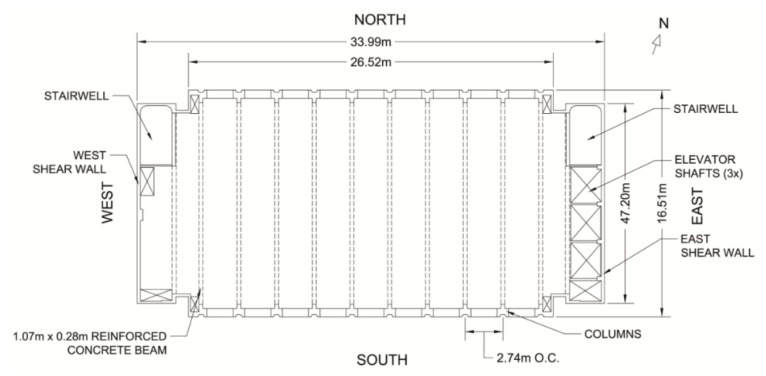
Plan view of a typical floor in the Green Building.

The Green Building is a 21 story academic building located on the campus of the Massachusetts Institute of Technology (MIT) in Cambridge (MA, USA; Figure 1). It is home to the lab, office, and classroom space of the Department of Earth and Planetary Sciences. The Green Building is a 83.7 m tall reinforced concrete building with a footprint of 16.5 m by 34 m (Figure 2). The short edges of the building are aligned at about 25° north-west. Henceforth, the short and long directions of the Green Building will be referred to as North-South and East-West (NS and EW). The first floor is 10 m above grade and houses a large lecture hall. The inter-story height of the first two floors is about 7.8 m, while that of the remaining floors is 3.5 m. Mechanical rooms are located on the top two floors, and heavy meteorological and radio equipment is asymmetrically mounted on the roof. Three elevator shafts are located on the eastern side of the building, while the building’s two stairwells are placed symmetrically at the North-East and North-West corners (Figure 2). The elevator shafts insert voids, measuring about 9.75 m by 2.21 m into the otherwise continuous floor slabs. The Eastern and Western facades are composed of 0.25 m thick shear walls, and floor slabs are typically 0.1016 m thick. The foundation consists of footings and circular piles with pile caps. The site is adjacent to the Charles River Basin, and it is primarily composed of fill material. Geotechnical investigations of the area have found the fundamental site frequency to be about 1.5 Hz [24].

### 2.1. Green Building Accelerometer Array

The Green Building is instrumented with 36 uniaxial EpiSensor ES-US force balance accelerometers, which sample at a rate of 200 Hz across a recording range of ±4 g. These are designed for structural monitoring purposes and produced by Kinemetrics [26]. The accelerometers are cable-connected to a central data recording station and time-synchronized using the global positioning system (GPS) with accuracy to within 1 ms. The data recorder is the Granite model, also produced by Kinemetrics [26]. The system is internet-connected and set to trigger if accelerations over 1 gal (0.01 m/s^2^) are detected. Acceleration data can also be collected through on-demand recordings and real-time streaming.

**Figure 3:**
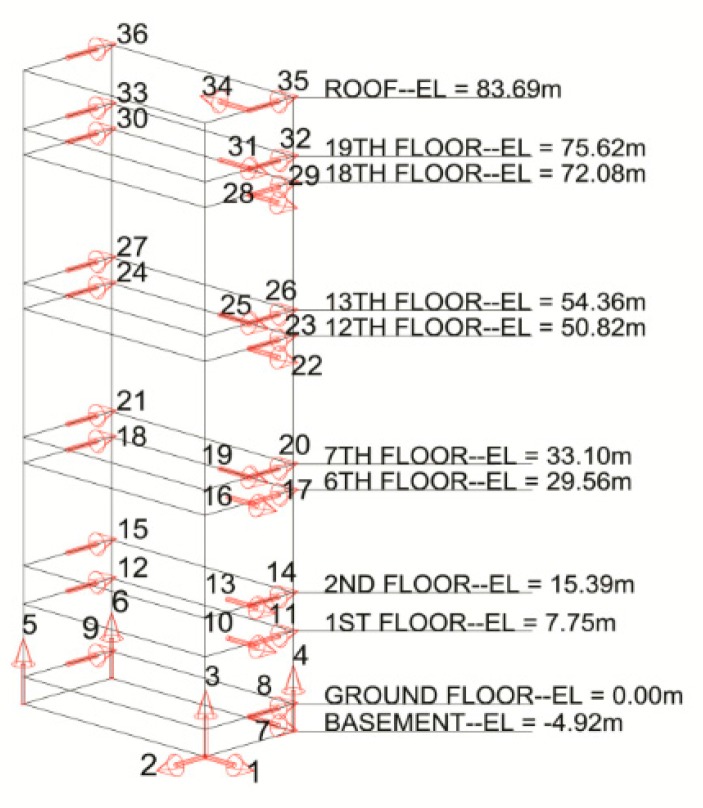
Distribution and orientation of USGS accelerometers within the Green Building.

The accelerometer placement and orientations are shown in Figure 3. This array and data recording system have been in operation since October 2010. The array is designed to monitor translations in the NS and EW directions, torsion, and base rocking motion. Torsional behavior is measured using parallel, NS-oriented sensors located at opposite ends of instrumented floors, while base rocking is determined using the four vertically (VT) aligned accelerometers in the basement. The sensor system continuously records building motion and stores acceleration data in an adjustable buffer for 5 days. Accelerometer data is collected in 1 second packets and output as raw ADC counts across a 24 bit range. The installed accelerometers operate on ±2.5 volt range and a sensitivity of 0.625 V/g. At least 10–15 min of vibration recording may provide better accuracy in calculation of modal properties, even though we did not measure all vibrational events with that duration [27].

### 2.2. Collected Data0.01225

Five sets of Green Building acceleration data were collected. Of these, two sets are of ambient vibrations and three are of strong excitations. This data was analyzed to characterize the Green Building’s dynamic behavior and structural characteristics and to validate a numerical model of the Green Building. The collected data is summarized in Table 1. All times are given according to the 24 h clock. The peak recorded accelerations generally increased with excitation in the following order: ambient vibrations (4/7/2011), ambient vibrations (6/22/2012), 4th of July fireworks show (7/4/2012), May 14 event (5/14/2012), and Hollis Center earthquake (10/16/2012). The May 14 and 4th of July fireworks induced the most high-frequency content in the Green Building. The lower structural translational and torsional modes were all found to be within the 0–10 Hz band.

#### 2.2.1. Ambient Vibrations (4/7/2011 and 6/22/2012)

**Figure 4:**
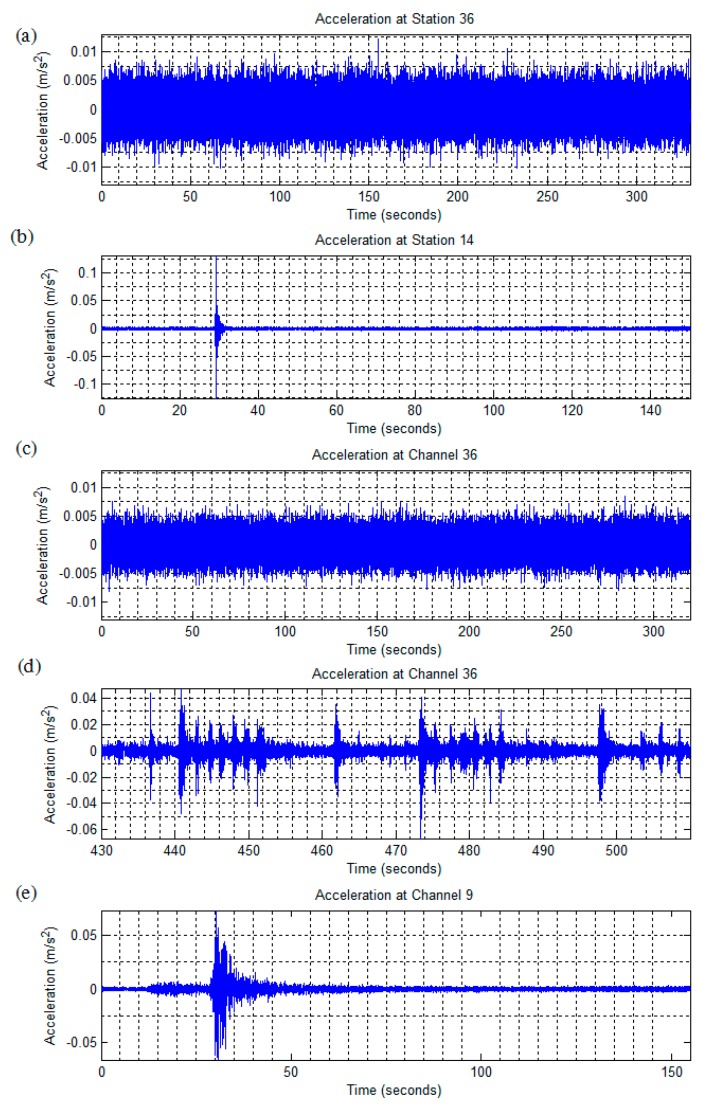
Sample time histories of recorded vibrations: (**a**) 4/7/2011 measurement: Acceleration time-history at the top floor (NS direction); (**b**) 5/14/2012 measurement: Acceleration time-history at the 2nd floor (NS direction); (**c**) 6/22/2012 measurement: Acceleration time-history at the top floor (NS direction); (**d**) 7/4/2012 measurement: Acceleration time-history at the top floor (NS direction) 10/16/2012 measurement: Response at the ground-level in the NS direction.

Two recordings of the Green Building’s ambient motions were made, one 330 second recording from Thursday 7 April, 2011 at 13:30:28 and another 320 second long recording from Friday 22 June, 2012 at 00:11:16. No seismic activity was reported during either recording and weather conditions were calm. A representative time history from roof level (Channel 36, east side, NS direction) is presented in Figure 4c.

#### 2.2.2. Unidentified Event: 5/14/2012

This unidentified event was first detected at the lower floors, and the eastern NS accelerometers reported higher peak accelerations than their counterparts along the western shear wall. The EW oriented accelerometers consistently measured higher accelerations than those along the NS axis. This suggests a ground-based shockwave very near the building’s eastern side. During this event, no seismic activity was recorded in the area, and a survey of the local news found no reports of explosions or other activities. Weather conditions were clear and calm with wind speeds of 5 mph out of the south. A time history is presented in Figure 4b.

#### 2.2.3. July 4th Fireworks Show: (7/4/2012)

An on-demand recording was made of the “Boston Pops Fireworks Spectacular” fireworks show, occurring on Wednesday July 4th, 2012. Weather conditions were rainy with sustained wind speeds of 3 mph from the West and wind gusts of 9 mph. There was negligible vehicle traffic; however, the area experienced extensive pedestrian activity throughout the evening. Fireworks were launched off barges on the Charles River, between the Harvard and Longfellow bridges, and about 0.6 km away from the Green Building. A representative acceleration time-history with a detail of the fireworks, recorded at the roof level in the NS direction (accelerometer channel 36) is presented in Figure 4d.

#### 2.2.4. Hollis Center Maine Earthquake: 10/16/2012

Acceleration response data was collected via an automatically triggered recording set off by an earthquake near Hollis Center (ME, USA), on Tuesday October 16, 2012. The recording began at 19:12:35 EST, and lasted 155 seconds. Weather conditions were clear, with negligible wind. The earthquake was of 4.0 magnitude, and the epicenter was located about 4 km outside of Hollis Center, which is located about 142 km from Cambridge (MA). A time-history of the event recorded at the Green Building’s ground level in the NS direction (accelerometer channel 9) is presented in Figure 4e.

### 2.3. Analyses of Dynamic Characteristics Using Field Measurements

The natural frequencies of the Green Building corresponding to motions in the EW, NS, and torsional (TR) directions are identified by visual inspection of the Fourier spectra, coherence spectra, and phase angles from the collected acceleration data. The fundamental modes are distinguishable in the ambient recordings, while higher modes occurred more clearly in the firework and 14 May event data sets.

Natural frequencies were identified by finding periodicities in the acceleration recordings using the Fourier and power spectra of the recordings, and then comparing signals from other accelerometers to determine the directionality of motion. Coherence spectra and phase angles between spatially separate accelerometers are checked to confirm building motions and to help estimate mode shapes. In total, six natural frequencies are identified (Table 2). To reduce redundancy, only a selection of the computed results is presented in the sections below.

#### 2.3.1. First Modes

The first EW, NS, and torsional (TR) modes are identified using ambient vibration measurements. As summarized in Table 2, the frequencies of these modes are found to be 0.68, 0.74, and 1.47 Hz, respectively. The spectra are taken at two floors in both the EW and NS directions. A peak at about 1.47 Hz is present in many channels, while peaks at 0.68 and 0.74 occur in only in the EW and NS directions, respectively. These peaks are found in both the July 4th firework (7/4/2012) and 10/16/2012 Hollis Center Maine earthquake event recordings. This suggests that these frequencies correspond to structural modes.

**Figure 5:**
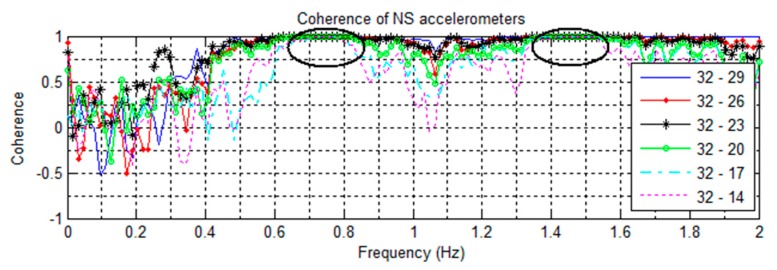
Coherences of NS oriented accelerometers paired against the floor 19 sensor; from the 6/22/2012 ambient vibration. Note the positive coherence at 0.74 Hz and 1.47 Hz.

**Figure 6:**
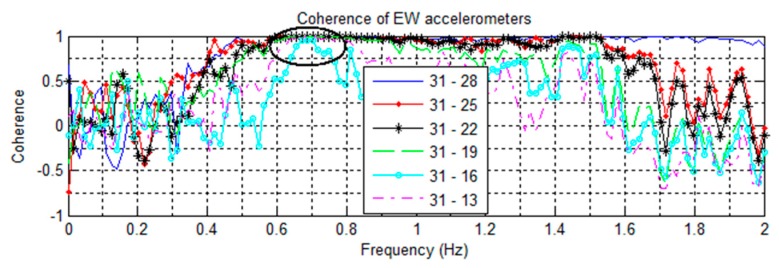
Coherences of EW oriented accelerometers paired against the floor 19 sensor; from the 6/22/2012 ambient vibration. Note the positive coherence at 0.68 Hz.

**Figure 7:**
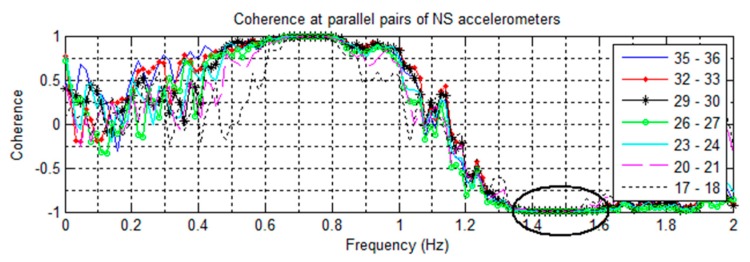
Coherences of parallel pairs of NS oriented accelerometers from the 6/22 ambient vibration recording. The negative coherence at 1.47 Hz indicates a torsional mode.

To determine the motion associated with these frequencies, the signal coherences are taken between pairs of accelerometers. Figure 5 shows the pairing of NS oriented accelerometers against the NS sensor (channel 32) at floor 19, and Figure 6 shows the same for accelerometers in the EW direction. This is done to establish whether the sensors are moving together as in a fundamental mode. To check for torsional motions, the coherences are computed between parallel NS oriented accelerometers located on the same floors (Figure 7).

In Figure 5, all the NS sensor pairings at 0.74 Hz show a very strong positive coherence of +1. Likewise, in Figure 6, the EW sensor pairings at 0.68 Hz show strong positive coherence. This shows the motions to be synchronous and corresponding to the fundamental NS and EW modes. In Figure 7, all pairings of parallel sensors show strong negative coherence at 1.47 Hz, indicating that the sensors are moving in opposite directions. This corresponds to the twisting motion of the first torsional mode. In the plot, all the pairs also have strong positive coherence at 0.74 Hz over the height of the building, indicating that the building is swaying symmetrically in the NS direction.

#### 2.3.2. Second Modes

The second EW, NS, and TR modes are found to be at 2.51, 2.81 and 5.05 Hz (Table 2). These modes appear in all recorded signals except the 10/16/2012 Hollis Center Maine earthquake, but they are strongest in the July 4th fireworks and the 14 May event recordings. This is due to the greater excitation energy needed to mobilize higher modes. The acceleration Fourier spectra of two floors in the NS and EW directions from the 14 May event and the 6/22/2012 ambient vibration measurements are calculated. In both spectra, the peak at 5.05 Hz is found, suggesting a whole-building motion, corresponding to a torsional mode. Conversely, the 2.51 and 2.81 Hz peaks only appear in EW and NS spectra.

**Figure 8:**
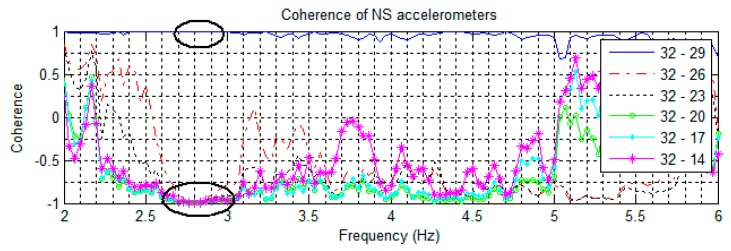
Coherences of NS oriented accelerometers paired against the floor 19 sensor. Note coherences at 2.81 Hz suggesting a cross-over point.

**Figure 9:**
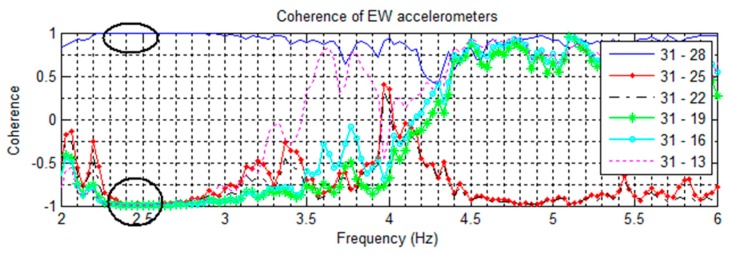
Coherences of EW oriented accelerometers paired against the floor 19 sensor. Note coherences at 2.51 Hz suggesting a cross-over point, and the coherence separation at 5.05 Hz.

**Figure 10:**
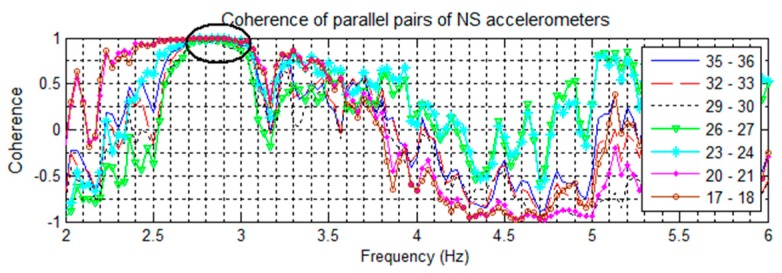
Coherences of parallel pairs of NS oriented accelerometers from the 14 May event. The strong positive coherence at 2.81 Hz suggests a NS mode.

Signal coherences of pairs of NS accelerometers are shown in Figure 8, Figure 9 contains EW pairings, and Figure 10 compares parallel NS oriented accelerometers. In Figure 8, the coherence at 2.81 Hz in the channel 32–29 pairing is nearly +1 coherent; while the rest of the channels paired with 32 are nearly −1 coherent, indicating that channels 32 and 29 are moving in the same direction while the other NS channels are moving in opposite directions. This suggests a mode shape nodal point located between floors 13 and 18. Furthermore, in Figure 10 all NS accelerometers on the same floor have a strong positive coherence. Both of these features indicate the 2nd NS mode to be at 2.81 Hz. In Figure 9, a similar split occurs in the coherences at 2.51 Hz, with channels 29–31 strongly positive while the rest are strongly negative, indicating a second EW mode. The cross-over point of the EW second mode shape also appears to be between floors 13 and 18.

For the second torsional mode, the 5.05 Hz frequency is present in all directions and all recorded excitations, suggesting a structural frequency. Figure 10 plots the parallel NS coherences and shows a trend towards negative coherence, while Figure 9 shows separation of coherences between certain EW sensors. Some separation is also visible with the NS sensors (Figure 8). In torsional motion, it is expected for some sensors to mover together while others move in opposite directions. For the EW sensors, the pairs of 25–31 and 22–31 are negatively coherent, while the remaining sensors have positive coherence. In both EW and NS cases, this corresponds to the region between floors 8 and 17. This matches the expected motion for the 2nd torsional mode, and the cross-over region is the same as that for the two second translational modes. 

All natural frequencies detected using coherence spectra analysis have broad margin as shown in Figure 5, Figure 6, Figure 7, Figure 8, Figure 9 and Figure 10. In order to improve the accuracy of the calculated modal properties, operational modal analysis (OMA) methods such as the enhanced frequency domain decomposition (EFDD) in the frequency domain and the stochastic subspace identification (SSI) in the time domain can be considered to analyze the experimental data more accurately ([17,28]).

#### 2.3.3. Torsional Behavior

**Figure 11:**
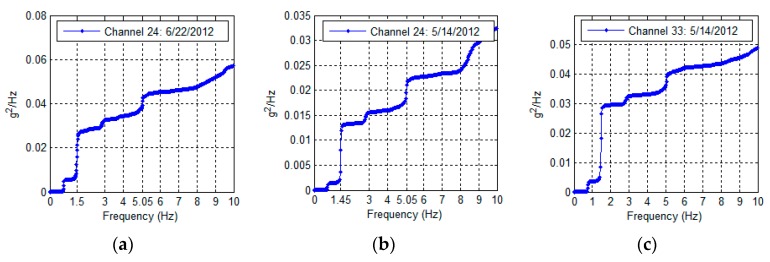
Cumulative spectral power at floor 12 during 22 June ambient vibrations (**a**) and the 14 May event (**b**) and cumulative spectral power at floor 19 during the 14 May event (**c**).

The Green Building is found to exhibit strong torsional behavior. The 1.47 Hz peak associated with the first torsional mode, and to a lesser extent the second torsional mode (5.05 Hz) is strongly and consistently represented in the building’s frequency content. An example of this is found in Figure 11 (left and middle), which shows the cumulative spectrum power of accelerations at floor 12 during ambient vibrations and the 5/14/2011 event. The torsional frequencies (1.47 and 5.05 Hz) are associated with the largest jumps in power content of the recorded motions. Figure 11 (right), shows the majority of spectral power at floor 19 to be in the fundamental torsional mode.

### 2.4. Foundation Rocking Motion

**Figure 12:**
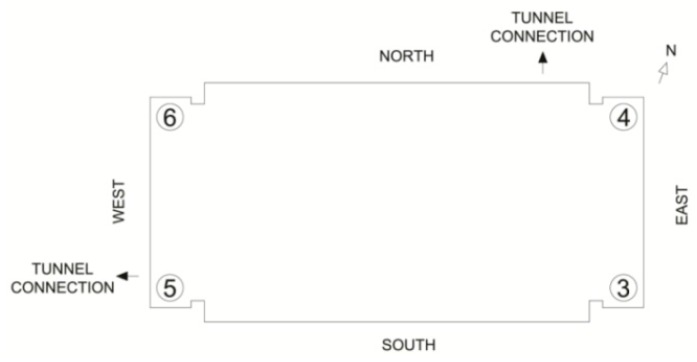
Distribution of vertical accelerometers in the Green Building’s basement level.

**Figure 13:**
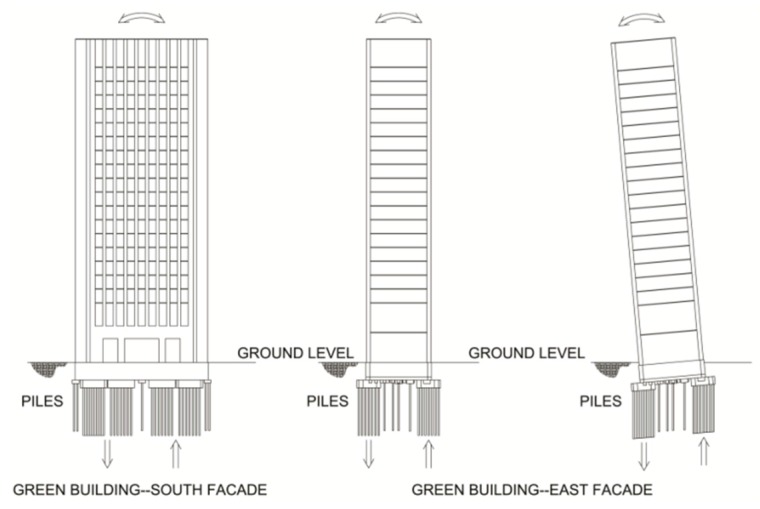
Base Rocking in the Green Building; along the NS and EW axis (**Left** and **Middle**). Rocking consists of a rigid-body rotation (exaggerated) and pile uplift and sinking (**Right**).

**Figure 14:**
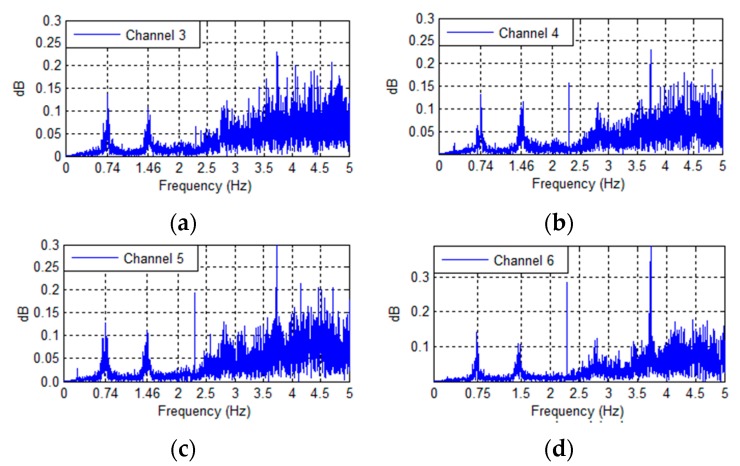
Fourier frequency spectra of the vertical base accelerometers subjected to the 4th of July fireworks show excitation (**a**) Channel 3; (**b**) Channel 4; (**c**) Channel 5; (**d**) Channel 6.

**Figure 15:**
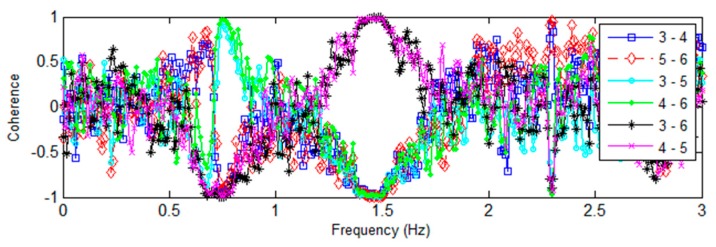
Coherence between vertically-aligned accelerometers in the basement of the Green Building subjected to the 4th of July fireworks show.

Base rocking behavior is determined by examining the frequency spectra, coherences, and phase angles of the vertically aligned accelerometers located in the basement level of the Green Building (Figure 12). Base rocking is a form of soil-structure interaction, where the foundation of a building undergoes a rigid-body rotation (Figure 13). The behavior of the vertical accelerometers is investigated around the lower natural frequencies, since differential base motion is expected to be coupled with the building’s torsional and translational modes. The Fourier spectra of the four vertical base accelerometers (Channels 3 to 6) are found in Figure 14. Peaks at 0.74, and 1.48 Hz, (the fundamental EW and torsional modes) are clearly identifiable. This confirms some coupling of base motions with the fundamental translational modes. Figure 15 presents the coherence between the vertical accelerometers in the 0–3 Hz range. Accelerometers located on the same North/South façade of the building (3–5 and 4–6) are positively coherent, while those on opposite or diagonal sides (3–4, 5–6, 3–6, and 4–5) have negative coherence. This implies that sensor pairs on the same North/South side are moving vertically together, while those on opposite sides are moving in opposite directions. Such motions correspond to a rotation of the base in the NS direction (about the building’s EW axis) which is coupled with the fundamental NS mode.

**Figure 16:**
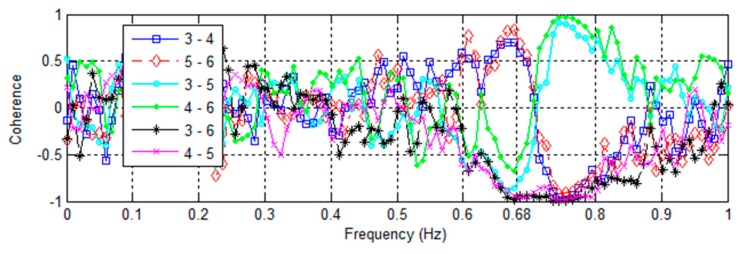
Coherence between vertically-aligned accelerometers in the basement of the Green Building subjected to the 4th of July fireworks show.

A detail of Figure 15 is shown in Figure 16. At 0.68 Hz, accelerometers on the same East or West façade of the building (3–4 and 5–6) have positive coherence, while those on opposite or diagonal sides (3–5, 4–6, 3–6, and 4–5) are negatively coherent. While there is some base rocking about the NS axis associated with the EW fundamental mode, the coupling and motion is not very strong. This corresponds to expectations, since the EW axis is in the building’s long direction, and significant base rocking would need to displace the foundation piles larger distances. Accelerometers on opposite diagonals of the base (3–6 and 4–5) are highly coherent while all others are strongly negative. This synchronous movement of opposite corners is expected of the torsional response in prismatic buildings, and indicates foundation flexure coupled with the fundamental torsional mode.

#### 2.4.1 East-West Asymmetries

**Figure 17:**
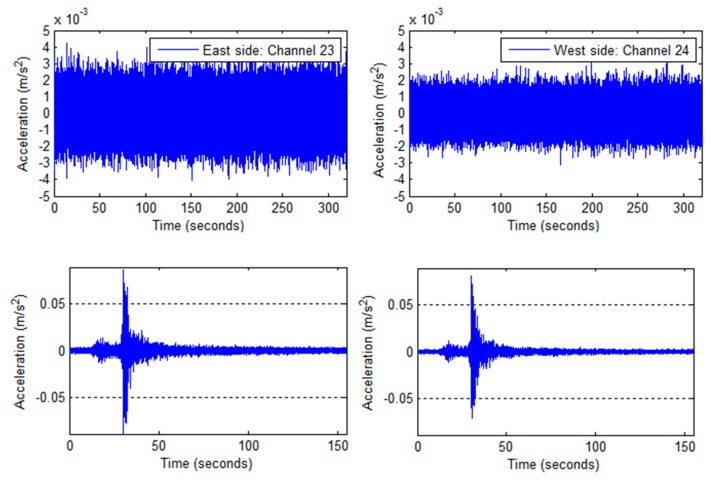
Accelerations from ambient vibrations (**Top**) and the Hollis Center earthquake (**Bottom**) at floor 12. Recordings from NS oriented accelerometers on the East side (**Left**) and West side (**Right**).

**Figure 18:**
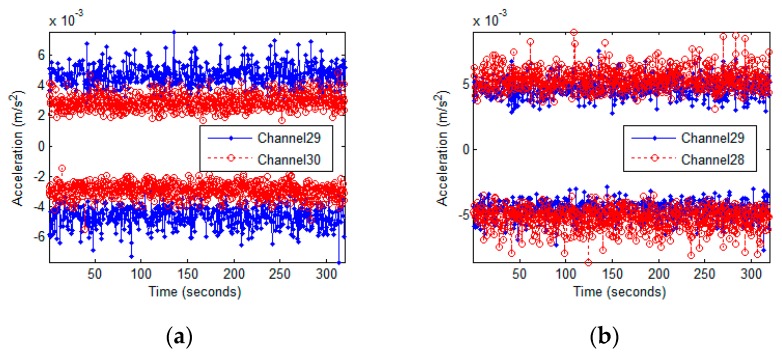
Noise levels at floor 18 due to ambient excitation. NS oriented accelerometers at opposite sides of the floor (**a**) and NS and EW oriented accelerometers at the eastern side (**b**).

Another feature observed in the acceleration data is an asymmetry in the dynamic behavior of the eastern and western sides of the Green Building. All three elevator shafts and their associated void space are located on the eastern side, while the two stairwells are in the northwest and northeast corners. Furthermore, heavy meteorological equipment is unevenly distributed on the roof. These asymmetries encourage twisting movement between the eastern and western sides. This feature is found by comparing the motions of parallel pairs of NS oriented accelerometers located on the same floors. The eastern side generally undergoes higher accelerations and has noisier signals. Figure 17 shows the time histories at the eastern and western sides of floor 12, subjected to ambient vibrations and the 10/16/2012 Hollis Center earthquake. The eastern side has higher accelerations due to both excitations. Figure 18 superimposes the noise envelope from ambient vibrations at floor 18. Higher noise levels are apparent on the eastern side. Higher ambient noise levels are also found in EW oriented accelerometers compared to their NS counterparts. Finally, Table 3 compares the peak accelerations between the eastern and western sides of the Green Building at floor 18. During the 14 May event, the eastern side experienced accelerations 1.42 times higher than the western side.

The peak vertical accelerations also indicate an asymmetry between the instrumented corners. The peak displacements of the vertical stations are presented in Table 4. Channel 5, at the foundation’s south-west corner, consistently experiences the highest displacements. Since a tunnel connection is at that location, this can be the result of foot and maintenance traffic causing more vibration noise than at the other locations. It could also be due to an issue with the tunnel connection, or stem from several piles in the south-west group being reported as either “lost” or having a “torn shell buried” in the as-built drawings.

## 3. Simplified Lumped-Mass Model Development

### 3.1. Modeling Procedure

In order to predict global dynamic behavior of the Green Building, a 3-D simplified lumped-mass stick model (SLMM) is developed. The SLMM models each floor as a point mass with 6 degrees of freedom—three transverse and three rotational. Successive floor point masses are joined by single beam elements which represent the equivalent stiffness of connecting walls and columns. To determine the equivalent beam stiffness of each story, the 6 × 6 stiffness and mass matrices are computed for each story. The 6 × 6 stiffness matrix is determined by condensing the distributed stiffness properties of an entire floor down to a single point. The procedures to develop a SLMM for tall buildings are as follows:

1. Develop a detailed finite element model of each unique story.
2. Extract node locations and the global stiffness matrix from the detailed FE model.
3. Condense the global stiffness matrix to the slab degrees of freedom using static condensation.
4. Condense the slab stiffness matrix down to a 6 × 6 stiffness matrix corresponding to a point on the slab surface by using geometric transformations.

Assemble each story’s 6 × 6 stiffness and mass matrices to develop the simplified stiffness and mass matrices for the entire building.

#### 3.1.1. Stiffness Matrix Estimation

Mass and stiffness matrices are extracted to develop 6 × 6 condensed matrices for simplified stick dynamic model of the 21-story building from the detailed finite element model (FEM) of each unique story. The detailed FEM of each story has fixed end boundary conditions in each end of the columns. Then each story has following procedures to develop condensed matrices. These condensed matrices in each story will be combined to each other to develop entire structure’s mass and stiffness matrices. In each story all nodes are divided into master or slave nodes. The slab nodes are assumed to drive the motion of the floor and they are defined as master nodes for static condensation to the floor slab DOFs. The remaining nodes are defined as slave nodes with zero mass. The dynamic equations of motion for an undamped system, excluding damping, are written in partitioned form, where master degrees of freedom have the ‘MM’ subscript.

(1) $$\left[\begin{array}{cc} M_{MM} & 0_{MS}\\ 0_{SM} & 0_{SS} \end{array}\right]\left\{\begin{array}{c} {\ddot{u}}_{M}\\ {\ddot{u}}_{S} \end{array}\right\}+\left[\begin{array}{cc} K_{MM} & K_{MS}\\ K_{SM} & K_{SS} \end{array}\right]\left\{\begin{array}{c} u_{M}\\ u_{S} \end{array}\right\}=\left\{\begin{array}{c} P_{M}\\ P_{S} \end{array}\right\}$$

$P_{S}$ is zero because no external forces are applied to the slave DOFs, $u_{S}$ denotes the slave DOFs, and $u_{M}$ denotes the master DOFs. Thus, Equation (1) is divided into two equations:

(2) $$M_{MM}{\ddot{u}}_{M}+K_{MM}u_{M}+K_{MS}u_{S}=P_{M}$$

(3) $$K_{SM}u_{M}+K_{SS}u_{S}=0$$

Equation (3) permits a static relationship between $u_{S}$ and $u_{M}$ because no inertia terms or external force are associated with $u_{S},$

(4) $$u_{S}=-K_{SS}^{-1}K_{SM}u_{M}$$

Then by substituting Equation (4) into Equation (2):

(5) $$M_{MM}{\ddot{u}}_{M}+K_{MM}u_{M}+K_{MS}(-K_{SS}^{-1}K_{SM}u_{M})=P_{M}$$

(6) $$M_{MM}{\ddot{u}}_{M}+(K_{MM}-K_{MS}K_{SS}^{-1}K_{SM})u_{M}=P_{M}$$

Then $K_{slab\;}=\;K_{MM}-K_{MS}K_{SS}^{-1}K_{SM}.$

The next step is to condense the slab degrees of freedom to a point on the slab surface which is considered as a rigid plate. Assuming that the N slab master nodes have six degrees of freedom:

(7) $$u_{M}={\left\{\begin{array}{cccc} u_{1} & u_{2} & \cdots & u_{N} \end{array}\right\}}^{T}$$

(8) $$u_{j}={\left\{\begin{array}{cccccc} u_{xj} & u_{yj} & u_{zj} & r_{xj} & r_{yj} & r_{zj} \end{array}\right\}}^{T}$$

(9) $$x_{j}={\left\{\begin{array}{ccc} x_{j} & y_{j} & z_{j} \end{array}\right\}}^{T}$$

where $u_{xj}$, $u_{yj}$,$u_{zj}$, $r_{xj}$, $r_{yj}$, and $r_{zj}$ are the three translational and three rotational displacements of node *j*, and $x_{j}$ $y_{j}$ $z_{j}$ are the coordinate of that node. A reference point, usually the center of mass, is defined as $x_{0}={\left\{\begin{array}{ccc} x_{0} & y_{0} & z_{0} \end{array}\right\}}^{T}$. The rigid body motion of the floor slab is:

(10) $$u_{M}=Tu_{0}$$

where $u_{0}={\left\{\begin{array}{cccccc} u_{x0} & u_{y0} & u_{z0} & r_{x0} & r_{y0} & r_{z0} \end{array}\right\}}^{T}$ are the rigid body motion components at reference point $x_{0}$, and the rigid body transformation matrix is:

(11) $$T=\left\{\begin{array}{c} T_{1}\\ T_{2}\\ \vdots \\ T_{N} \end{array}\right\},\;T_{j}=\left[\begin{array}{cccccc} 1 & 0 & 0 & 0 & dz_{j} & -dy_{j}\\ 0 & 1 & 0 & dz_{j} & 0 & dx_{j}\\ 0 & 0 & 1 & dy_{j} & -dx_{j} & 0\\ 0 & 0 & 0 & 1 & 0 & 0\\ 0 & 0 & 0 & 0 & 1 & 0\\ 0 & 0 & 0 & 0 & 0 & 1 \end{array}\right],\text{ where }(\begin{array}{c} dx_{j}=x_{j}-x_{0}\\ dy_{j}=y_{j}-y_{0}\\ dz_{j}=z_{j}-z_{0} \end{array}$$

For simplicity, the reference point of each story is taken to be at elevation $z_{0}$ = 0, so all $dz_{j}$ terms are 0. z coordinate of each slab node is zero. The rotational effects due to the distances $dx_{j}$ and $dy_{j}$ are effected to center of geometry’s global Z-directional rotation. Hence:

(12) $$p_{M}=K_{slab}u_{M}=K_{slab}Tu_{0}$$

Next, we assume that external forces are applied only to the master nodes. The external forces applied to the master node are related to the forces on all remnant nodes by the following transformation:

(13) $$p_{0}=T^{T}p_{M},\;p_{0}=\left\{\begin{array}{cccccc} p_{x0} & p_{y0} & p_{z0} & M_{x0} & M_{y0} & M_{z0} \end{array}\right\}$$

Combining Equations (12) and (13):

(14) $$p_{0}=T^{T}K_{slab}Tu_{0}=\tilde{K}u_{0}$$

where $\tilde{K}=T^{T}K_{slab}T$. $\tilde{K}$ is the story’s 6 × 6 stiffness matrix.

The 6 × 6 stiffness matrix of each floor can now be transformed into the 12 × 12 stiffness of a standard beam element. $\hat{K}$ is the stiffness matrix of a beam element whose displacements or degrees of freedom $u_{i},$ $u_{j}$ are defined at the two ends such as first story and second story of the building. i, and j are the specific story numbers with coordinates $x_{i},$ and $x_{j}.$ The stiffness matrix of this beam element will have the form:

(15) $$\hat{K}=\left\{\begin{array}{cc} {\tilde{K}}_{ii} & {\tilde{K}}_{ij}\\ {\tilde{K}}_{ji} & {\tilde{K}}_{ii} \end{array}\right\}$$

${\tilde{K}}_{ii}$, and ${\tilde{K}}_{jj}$ are stiffness matrices of the rigid plate relative to any one end, j, which consists of three translations and three rotations. The displacements at the other end are then defined as:

(16) $$u_{i}=T_{ij}u_{j}$$

(17) $$T_{ij}=\left[\begin{array}{cccccc} 1 & 0 & 0 & 0 & dz_{ij} & -dy_{ij}\\ 0 & 1 & 0 & dz_{ij} & 0 & dx_{ij}\\ 0 & 0 & 1 & dy_{ij} & -dx_{ij} & 0\\ 0 & 0 & 0 & 1 & 0 & 0\\ 0 & 0 & 0 & 0 & 1 & 0\\ 0 & 0 & 0 & 0 & 0 & 1 \end{array}\right],\text{ where }(\begin{array}{c} dx_{ij}=x_{i}-x_{j}\\ dy_{ij}=y_{i}-y_{j}\\ dz_{ij}=z_{i}-z_{j} \end{array}$$

For an upright beam element, $dx_{ij}$ and $dy_{ij}$ are zero, and $dz_{ij}$ is height of each story. A rigid body motion of a free body requires no external forces, then:

(18) $$\left\{\begin{array}{cc} {\tilde{K}}_{ii} & {\tilde{K}}_{ij}\\ {\tilde{K}}_{ji} & {\tilde{K}}_{jj} \end{array}\right\}\left\{\begin{array}{c} T_{ij}\\ I \end{array}\right\}=\left\{\begin{array}{c} 0\\ 0 \end{array}\right\}$$

Hence:

(19) $${\tilde{K}}_{ij}=-{\tilde{K}}_{ii}^{T}T_{ij}$$

(20) $${\tilde{K}}_{ji}={\tilde{K}}_{ij}^{T}=-T_{ij}^{T}{\tilde{K}}_{ii}^{T}=-T_{ij}^{T}{\tilde{K}}_{ii}$$

where ${\tilde{K}}_{jj}={\tilde{K}}_{ji}T_{ij}^{T}$ and ${\tilde{K}}_{jj}=T_{ij}^{T}{\tilde{K}}_{ii}T_{ij}$. Thus, the entire stiffness matrix for N number of story building is expressed as:

(21) $$\hat{K}=\left[\begin{array}{ccccccc} {\tilde{K}}_{11}^{N} & {\tilde{K}}_{12}^{N} & 0 & 0 & 0 & 0 & 0\\ {\tilde{K}}_{21}^{N} & {\tilde{K}}_{22}^{N}+{\tilde{K}}_{11}^{N-1} & {\tilde{K}}_{12}^{N-1} & 0 & 0 & 0 & 0\\ 0 & {\tilde{K}}_{21}^{N-1} & {\tilde{K}}_{22}^{N-1}+{\tilde{K}}_{11}^{N-2} & {\tilde{K}}_{12}^{N-3} & 0 & 0 & 0\\ 0 & 0 & {\tilde{K}}_{21}^{N-2} & {\tilde{K}}_{22}^{N-3}+{\tilde{K}}_{11}^{N-4} & 0 & 0 & 0\\ 0 & 0 & 0 & {\tilde{K}}_{21}^{N-4} & \ddots & \vdots & \vdots \\ 0 & 0 & 0 & 0 & \ldots & {\tilde{K}}_{11}^{1} & {\tilde{K}}_{12}^{1}\\ 0 & 0 & 0 & 0 & \cdots & {\tilde{K}}_{21}^{1} & {\tilde{K}}_{22}^{1} \end{array}\right]$$

where N is the *N*-th number of story of the entire building. To apply the boundary conditions of the entire Green building model, the last row and column of the $\hat{K}$ are removed since the bottom columns are assumed to be fixed.

#### 3.1.2. Mass Matrix

Here is a necessary overview of the floor models we used. In summary, we used Autodesk Simulation Multiphysics (ASM) and modeled the floors using beam and shell elements. The 6 × 6 mass matrix consists of the total floor mass, the mass products of inertia, and the mass moments of inertia. These quantities can either be computed by hand based on the floor geometry, or output from ASM. The mass product and mass moment of inertia are computed relative to the center of mass (reference point).

The global mass matrix of the SLMM is assembled by overlapping the 12 × 12 mass matrices of each equivalent beam element. As with the stiffness matrix, only the 6 × 6 mass matrix of each story is needed to define each story’s full mass matrix. This 6 × 6 matrix is found by computing the mass properties of a floor and assuming that each of the two nodes defining the equivalent beam receives one half of the mass properties:

(22) $$J_{xx}=\int_{V}(y^{2}+z^{2})\rho dV$$

(23) $$J_{xy}=-\int_{V}xy\rho dV$$

(24) $$M^{N}=\left[\begin{array}{cccccc} m_{x} & 0 & 0 & 0 & 0 & 0\\ 0 & m_{y} & 0 & 0 & 0 & 0\\ 0 & 0 & m_{z} & 0 & 0 & 0\\ 0 & 0 & 0 & J_{xx} & J_{xy} & J_{xz}\\ 0 & 0 & 0 & J_{yx} & J_{yy} & J_{yz}\\ 0 & 0 & 0 & J_{zx} & J_{zy} & J_{zz} \end{array}\right]$$

The form of an individual floor mass matrix is found in Equation (24), where *m_x_* = *m_y_* = *m_z_* is the full floor mass; *J_xx_*, *J_yy_* and *J_zz_* are the mass moments of inertia with respect to a point aligned with the center of mass; and *J_xy_* = *J_yx_*, *J_xz_* = *J_zx_* and *J_yz_* = *J_zy_* are the mass products of inertia, with respect to the same point. Given a set of orthogonal axis *u*, *v* and *w* and a material density *ρ*, the equations for the mass moments and mass products of inertia are given in Equations (22) and (23). In practice, the mass products are very small and can be neglected. Density of the concrete is taken to be 2400 kg/m^3^.

Using $M^{N}$ matrix of *N*-th of the Green Building in Equation (24), the final lumped mass matrix of the Green building is developed using the same procedure as for the stiffness matrix (Equation (21)). The lumped global mass matrix of the entire Green building is:

(25) $$\hat{M}=\left[\begin{array}{cccccc} M^{N} & 0 & 0 & 0 & 0 & 0\\ 0 & M^{N}/2+M^{N-1}/2 & 0 & 0 & 0 & 0\\ 0 & 0 & M^{N-1}/2+M^{N-2}/2 & 0 & 0 & 0\\ 0 & 0 & 0 & M^{N-2}/2+M^{N-3}/2 & 0 & 0\\ 0 & 0 & 0 & 0 & \ddots & \vdots \\ 0 & 0 & 0 & 0 & \cdots & M^{1}/2 \end{array}\right]$$

To get the mass matrix of the entire Green Building, the last row and column of $\hat{M}$ are removed due to the clamped boundary condition. Also $M^{N}/2$ is multiplied by two to consider additional mass from roof mounted radio and meteorological equipment.

#### 3.1.3. Damping Matrix

In order to develop the damping matrix of the structure using the mass and stiffness matrices, $\hat{M}$ and $\hat{K}$, Rayleigh proportional damping is used:

(26) $$\hat{C}=\alpha \hat{M}+\beta \hat{K}$$

where:

(27) $$\alpha =\frac{2\omega_{i}\omega_{j}(\xi \omega_{j}-\xi \omega_{i})}{\omega_{j}^{2}-\omega_{i}^{2}},\;\beta =\frac{2(\xi \omega_{j}-\xi \omega_{i})}{\omega_{j}^{2}-\omega_{i}^{2}}$$

where ξ is a 3% damping ratio, and $\omega_{i}$ and $\omega_{j}$ are natural frequencies where i and j are 1 and 2, respectively.

### 3.2. Modal and Time History Analysis

Modal updating is carried out to match the natural frequencies between the measured from sensors and the calculated from the developed SLMM. In order to find optimal mass and stiffness values in each story of the structure, advanced multi-objective genetic algorithms are applied [29,30]. The fitness objective functions of the optimization are:

(28) $$\begin{array}{l} J_{1}:\;\sqrt{\frac{\sum_{t=1}^{n}{\left({\text{channels}}_{\text{N-S and E-W directions},\text{t}}{-\text{time history}}_{\text{corresponding N-S and E-W directions},\text{t}}\right)}^{2}}{n}}\\ J_{2}:\;\sum_{f=1}^{6}{\text{Abs}(\text{natural frequencies}}_{measured,f}{-\text{natural frequencies}}_{calculated,f}) \end{array}$$

where, n is number of time steps in measurements and absolute displacement of the time history analysis, and f is number of natural frequencies measured and calculated. Single objective based composite objective function requires weight for each component of the objectives to evenly or purposely assign the contribution of each objective to an eventual fitness objective value. However, multi-objective optimization does not require those weights, which is one of the main advantages of multi-objective based optimization [31,32]. In order to carry out a time history analysis for the 10/16 Hollis Center Maine Earthquake, input earthquakes for EW- and NS directions are calculated using the recorded acceleration data from the following channels:

(29) $$\begin{array}{l} \text{For EW direction}=\text{Channel }7\\ \text{For NS direction}=\frac{\text{Channel }8+\text{Channel }9}{2} \end{array}$$

For the EW direction, Channel 7 is used because we do not have acceleration data from west facades of the building, and for NS direction average of the two related accelerations are used for the center of the mass because SLMM is a lumped mass model. In Equation (28) the root-mean-square errors are also calculated using the average of the two related displacements. Newmark-β time-step integration method has been used in the simulation for the SLMM. The incremental governing equations of motion for the structural system are expressed as:

(30) $$\hat{M}\Delta \ddot{U}+\hat{C}\Delta \dot{U}+\hat{K}\Delta U=-\hat{M}G\Delta {\ddot{x}}_{g}$$

where M, C, and K are reduced order mass, damping, and stiffness matrices of the 21-story building, ΔU is incremental response vector, and G, and $\Delta {\ddot{x}}_{g}$ are loading vector for the earthquakes, ground acceleration increment, respectively. 

In order to update each value of mass and stiffness matrices of each story, a vector of n physically significant parameters is to be updated:

(31) $$R=\left(\begin{array}{cccc} r_{1} & r_{2} & \cdots & r_{n} \end{array}\right)$$

in which *r_i_* is the *i-*th rate of the *i-*th parameter. For this structure, it has four parameters for each mass matrix (i.e., *m*_11_ = *m*_22_ = *m*_33_, *m*_44_, *m*_55_ and *m*_66_) and nine parameters for each stiffness matrix (i.e., *k*_11_, *k*_22_, *k*_33_, *k*_44_, *k*_55_, *k*_66_, *k*_24_ = *k*_42_, *k*_15_ = *k*_51_ and *k*_45_ = *k*_54_) of each story and total 273 parameters, which is n in Equation (31). The updated parameters from the multi-objective optimization is used to calculate new mass and stiffness values in each story as follows:

(32) $$\begin{array}{l} m_{ui}=\;m_{oi}+r_{i}m_{oi}\\ k_{ui}=\;k_{oi}-r_{i}k_{oi} \end{array}$$

where subscripts *ui*, and *oi* are the updated and original mass and stiffness values, respectively. Mass values are increased from the Equation (32) to consider nonstructural components and stiffness values are decreased due to damage and deterioration of materials.

In order to update modal properties, the natural frequencies from the earthquake ground motion (Maine) are used instead of the average values from various vibrations from the Table 2 for better synchronization in time histories of acceleration at top story. The 2nd torsional frequency, 5.05, is also taken from the average value because the frequency was not achieved from the earthquake as shown in Table 2. Updated structural stiffness and mass information are achieved as shown in Table 5 and Table 6 using the advanced multi-objective optimization in terms of measured natural frequencies and acceleration time history. The initial masses were underestimated in the SLMM because contributing live load and the superimposed dead load masses were not considered. In order to consider the mass of non-structural members such as interior partitions and any additional masses due to heavy experimental equipment, the increment of mass values in each component of mass matrix in each story is assigned as maximum 30% of the original self-weight with the resolution of 1%. The stiffness of each story is also updated with the reduction of a maximum of 50% of the original SLMM to consider degradation of stiffness due to strong winds and minor earthquakes during last 50 years, which is based on previous research done by Skolnik et al. [12]. The Green building was constructed in 1964.

The discrepancy of the natural frequencies between initial simplified FE model and measured data are significant as shown in Table 7. It is reasonable because the contributing live load and the superimposed dead load masses were not considered as we mentioned in the previous paragraph and simultaneously the stiffness of the structure has been gradually reduced during 50 years due to strong winds in Boston area and material degradation. Similar result was reported by Bradford et al. [33]; the natural frequencies of the Millikan Library building, which is also a concrete structure, at Caltech (Pasadena, CA, USA) have been reduced more than 30% during 30 years due to degradation of strength of materials and strong and minor earthquakes. From the updated stiffness values in Table 6, we found that the torsional stiffness values and coupling stiffness values are more reduced compared to NS or ES directional stiffness values, which may be due to exposure to strong winds during the last 50 years.

**Figure 19:**
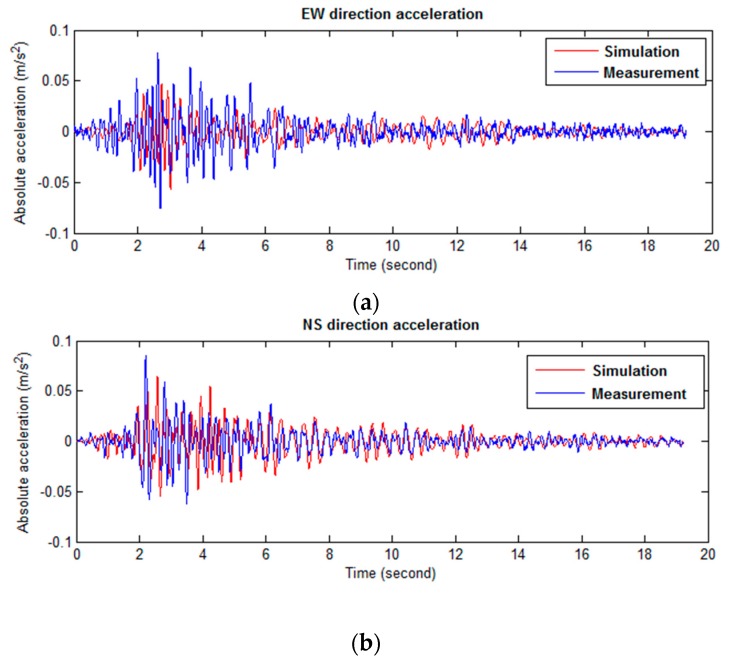
Time history comparison between predicted and measured roof accelerations for 10/16/2012 Hollis Center Maine earthquake (**a**) EW directional acceleration time history; (**b**) NS directional acceleration time history.

The predicted response of the updated SLMM to the 10/16 Hollis Center Maine earthquake in Table 7 is also compared to the measured vibration data, as shown in Figure 19. The overall responses show good agreement each other. Especially the NS directional responses are quite well matched with each other, even though it is the response of the top story which is the result of the accumulated responses of lower stories. It means that the discrepancies are also able to be accumulated. In terms of that viewpoint, the achieved result is quite satisfactory, but there are discrepancies with respect to the amplitude of the EW direction may be the result of the inaccurate earthquake input for that direction. The assumed damping ratios and simplified model, which is not able to consider higher modes of the building with accumulated errors due to high-rise building, may cause discrepancies in time history responses as shown in Figure 19. Thus, as future work, in order to more precisely predict structural behavior of the building, we will include parameters of damping ratios for better synchronization, nonlinear Kalman filtering based system identification method could be used to predict structural properties such as stiffness and damping for each DOF and also to predict structural responses when it is subjected to future random earthquake ground motions.

## 4. Conclusions

In order to identify dynamic characteristics of tall building systems, a sensor network with 36 accelerometers was used to measure accelerations within MIT’s 21 story Green Building, due to ambient vibrations, seismic loading, and firework excitations. The acceleration data collected using the accelerometer array was analyzed to determine the vibrational characteristics of the Green Building. The frequencies of six normal vibrational modes were identified, including East-West (EW) modes at 0.68 Hz and 2.51 Hz; North-South (NS) modes at 0.74 Hz and 2.81 Hz; and two torsional modes at 1.47 Hz and 5.05 Hz. For each mode, these frequencies stack in the order of EW, NS, and torsional. No vertical translational modes were observed. However, the building was found to strongly exhibit soil-structure interaction effects called base rocking and base flexure. Base rocking was coupled strongly with all three fundamental modes, and it was also associated with higher EW frequencies. Base flexure was observed to occur with the torsional modes. The base motion behavior is thought to be due to the geotechnical characteristics of the site and asymmetries in the Green Building’s pile foundation.

In general, the Green Building was observed to undergo strong torsional movement and to exhibit slightly different motions at its eastern and western ends. Torsional frequencies were represented in all the collected data sets, and their power often exceeded that of the translational modes. The eastern side of the building experienced higher accelerations and higher ambient noise levels. Both the strong torsional response and the differences in east-west behavior are thought to result from structural asymmetries, uneven dead load distribution (in the form of roof-mounted meteorological equipment and other machinery), and irregularities in the pile foundation. The site’s fundamental frequency of 1.48 Hz, which is close to the building’ fundamental torsional frequency of 1.47 Hz, also probably amplifies twisting motion. 

Using the original design documents, the Green Building was numerically modeled with a 3-D simplified lump-mass stick model. Modal updating was carried out by an advanced multiobjective optimization method using the measured structural responses and their modal properties. This updated model was validated by comparison of natural frequencies and seismic response with the collected acceleration data. Initial simulations of seismic excitations demonstrated the model to have good agreement with measured values. For future work, the numerical model will be enhanced to account for soil-structure interaction effects. The structural characterization of the Green Building will be used to further develop vibration-based damage detection methodologies and to predict structural performance during seismic events.

## Figures and Tables

**Table 1:** Summary of collected measurement data *.

Date		4/7/2011	5/14/2011	6/22/2012	7/4/2012	10/16/2012
Type		Ambient	Ext. event	Ambient	Fireworks	Earthquake
Peak Accel. (m/s^2^)	[NS]	0.01225 (0.0012g) {36}	0.1321 (0.0135g) {14}	0.01000 (0.0010g) {35}	0.0663 (0.0068g) {36}	0.1218 (0.0124g) {35}
	[EW]	0.02026 (0.0021g) {36}	0.6651 (0.0678g) {34}	0.02422 (0.0025g) {34}	0.1808 (0.0184g) {13}	0.1214 (0.0124g) {10}
	[VT]	0.001507 (0.0002g) {6}	0.0133 (0.0014g) {5}	0.001965 (0.0002g) {6}	0.0162 (0.0017g) {5}	0.0389 (0.0040g) {6}
Starting time		13:24:58	2:04:15	00:11:16	22:32:05	19:12:47
Duration (s)		330	150	320	510	155

* Numbers in braces refer to the acceleration channel number in Figure 3.

**Table 2:** Natural frequencies of the Building identified using data from the accelerometer array.

Data	4/7/2011		5/14/2012		6/22/2012		7/4/2012		10/16/2012		Mean (Hz)
Channel	31/32	22/23	31/32	22/23	31/32	22/23	31/32	22/23	31/32	22/23	
1st mode EW	0.69	0.69	0.69	0.69	0.70	0.70	0.67	0.67	0.66	0.66	**0.68**
1st mode NS	0.76	0.76	0.73	0.73	0.75	0.75	0.74	0.74	0.74	0.74	**0.74**
1st mode TR	1.46	1.46	1.45	1.45	1.50	1.51	1.46	1.46	N/A	1.45	**1.47**
2nd mode EW	2.56	2.64	2.51	2.51	2.55	2.62	2.48	2.46	2.39	2.39	**2.51**
2nd mode NS	2.83	2.83	2.77	2.77	2.82	2.82	2.81	2.81	2.81	2.81	**2.81**
2nd mode TR	5.14	4.99	5.05	5.04	5.04	5.04	5.04	5.03	N/A	N/A	**5.05**

**Table 3:** Comparison of peak accelerations between parallel NS oriented accelerometers at floor 18 due to various excitations.

Vibrations	East Side (m/s^2^)	West Side (m/s^2^)	Ratio
6/22 Ambient	0.0077	0.0055	1.39
May 14 Event	0.0457	0.0322	1.42
Hollis Center Maine Earthquake	0.0582	0.0420	1.39

**Table 4:** Peak displacements at the vertical accelerometers.

Vibrations	Peak Displacements (m)			
Channel	3	4	5	6
6/22 Ambient Vibration	5.79 × 10^−7^	6.12 × 10^−7^	1.36× 10^−6^	5.42 × 10^−7^
Fireworks	1.13 × 10^−6^	1.15 × 10^−6^	1.56 × 10^−6^	1.20 × 10^−6^
5/14 Event	9.37 × 10^−7^	5.88 × 10^−7^	1.57 × 10^−6^	5.78 × 10^−7^
10/16 Maine Earthquake	4.18 × 10^−5^	3.08 × 10^−5^	3.77 × 10^−6^	3.41 × 10^−5^

**Table 5:** Mass and stiffness properties of the Green building: unit (kg).

Floor	$m_{11}$	$m_{22}$	$m_{33}$	$m_{44}$	$m_{55}$	$m_{66}$
Initial (2nd:20th)	4.22 × 10^5^	4.22 × 10^5^	4.22 × 10^5^	1.46 × 10^7^	5.05 × 10^7^	6.33 × 10^7^
20th	5.49 × 10^5^	5.49 × 10^5^	5.49 × 10^5^	1.47 × 10^7^	6.11 × 10^7^	6.78 × 10^7^
19th	5.49 × 10^5^	5.49 × 10^5^	5.49 × 10^5^	1.57 × 10^7^	6.41 × 10^7^	6.33 × 10^7^
18th	5.49 × 10^5^	5.49 × 10^5^	5.49 × 10^5^	1.48 × 10^7^	6.26 × 10^7^	7.79 × 10^7^
17th	5.49 × 10^5^	5.49 × 10^5^	5.49 × 10^5^	1.50 × 10^7^	5.25 × 10^7^	7.91 × 10^7^
16th	5.49 × 10^5^	5.49 × 10^5^	5.49 × 10^5^	1.57 × 10^7^	5.25 × 10^7^	8.04 × 10^7^
15th	5.49 × 10^5^	5.49 × 10^5^	5.49 × 10^5^	1.47 × 10^7^	5.25 × 10^7^	6.46 × 10^7^
14th	5.49 × 10^5^	5.49 × 10^5^	5.49 × 10^5^	1.59 × 10^7^	6.06 × 10^7^	7.16 × 10^7^
13th	5.49 × 10^5^	5.49 × 10^5^	5.49 × 10^5^	1.75 × 10^7^	5.05 × 10^7^	7.79 × 10^7^
12th	5.41 × 10^5^	5.41 × 10^5^	5.41 × 10^5^	1.69 × 10^7^	5.45 × 10^7^	6.97 × 10^7^
11th	5.49 × 10^5^	5.49 × 10^5^	5.49 × 10^5^	1.51 × 10^7^	5.71 × 10^7^	7.66 × 10^7^
10th	5.41 × 10^5^	5.41 × 10^5^	5.41 × 10^5^	1.60 × 10^7^	6.46 × 10^7^	6.39 × 10^7^
9th	5.49 × 10^5^	5.49 × 10^5^	5.49 × 10^5^	1.66 × 10^7^	5.35 × 10^7^	7.09 × 10^7^
8th	4.22 × 10^5^	4.22 × 10^5^	4.22 × 10^5^	1.46 × 10^7^	5.05 × 10^7^	6.33 × 10^7^
7th	5.07 × 10^5^	5.07 × 10^5^	5.07 × 10^5^	1.75 × 10^7^	6.46 × 10^7^	7.03 × 10^7^
6th	5.03 × 10^5^	5.03 × 10^5^	5.03 × 10^5^	1.85 × 10^7^	5.30 × 10^7^	6.52 × 10^7^
5th	4.22 × 10^5^	4.22 × 10^5^	4.22 × 10^5^	1.56 × 10^7^	5.71 × 10^7^	8.23 × 10^7^
4th	4.22 × 10^5^	4.22 × 10^5^	4.22 × 10^5^	1.59 × 10^7^	5.96 × 10^7^	6.46 × 10^7^
3rd	4.22 × 10^5^	4.22 × 10^5^	4.22 × 10^5^	1.73 × 10^7^	5.40 × 10^7^	7.85 × 10^7^
2nd	4.22 × 10^5^	4.22 × 10^5^	4.22 × 10^5^	1.73 × 10^7^	6.26 × 10^7^	6.78 × 10^7^
Initial (1st)	7.61 × 10^5^	7.61 × 10^5^	7.61 × 10^5^	4.26 × 10^7^	1.04 × 10^8^	1.22 × 10^8^
1st	7.61 × 10^5^	7.61 × 10^5^	7.61 × 10^5^	4.43 × 10^7^	1.05 × 10^8^	1.33 × 10^8^
Initial(Ground)	7.50 × 10^5^	7.50 × 10^5^	7.50 × 10^5^	3.89 × 10^7^	1.08 × 10^8^	1.27 × 10^8^
Ground	7.57 × 10^5^	7.57 × 10^5^	7.57 × 10^5^	4.16 × 10^7^	1.14 × 10^8^	1.34 × 10^8^

**Table 6:** Stiffness properties of the Green building: unit (N/m).

Floor	$k_{11}$	$k_{22}$	$k_{33}$	$k_{44}$	$k_{55}$	$k_{66}$	$k_{24}=k_{42}$	$k_{15}=k_{51}$	$k_{45}=k_{54}$
Initial (2nd:20th)	1.79 × 10^10^	3.94 × 10^10^	2.59 × 10^11^	1.56 × 10^13^	4.06 × 10^13^	1.00 × 10^13^	7.31 × 10^10^	−3.32 × 10^10^	4.30 × 10^10^
20th	1.56 × 10^10^	3.86 × 10^10^	2.28 × 10^11^	7.82 × 10^13^	2.72 × 10^13^	5.21 × 10^12^	4.75 × 10^10^	−2.45 × 10^10^	3.96 × 10^10^
19th	1.41 × 10^10^	3.71 × 10^10^	1.30 × 10^11^	1.05 × 10^13^	2.31 × 10^13^	6.81 × 10^12^	6.14 × 10^10^	−2.82 × 10^10^	3.40 × 10^10^
18th	9.30 × 10^9^	3.51 × 10^10^	1.79 × 10^11^	1.35 × 10^13^	4.06 × 10^13^	7.91 × 10^12^	4.02 × 10^10^	−2.06 × 10^10^	3.57 × 10^10^
17th	1.00 × 10^10^	3.94 × 10^10^	2.05 × 10^11^	1.33 × 10^13^	3.81 × 10^13^	8.21 × 10^12^	7.31 × 10^10^	−3.28 × 10^10^	3.23 × 10^10^
16th	9.48 × 10^9^	3.08 × 10^10^	2.36× 10^11^	1.52 × 10^13^	2.23 × 10^13^	9.92 × 10^12^	5.77 × 10^10^	−3.02 × 10^10^	2.50 × 10^10^
15th	1.14 × 10^10^	3.55 × 10^10^	1.79 × 10^11^	1.38 × 10^13^	2.80 × 10^13^	9.42 × 10^12^	4.17 × 10^10^	−3.22 × 10^10^	3.19 × 10^10^
14th	1.00 × 10^10^	3.19 × 10^10^	2.54 × 10^11^	1.41 × 10^13^	2.68 × 10^13^	5.91 × 10^12^	4.24 × 10^10^	−1.66 × 10^10^	2.45 × 10^10^
13th	9.30 × 10^9^	2.84 × 10^10^	1.95 × 10^11^	1.52 × 10^13^	2.39 × 10^13^	7.31 × 10^12^	5.48 × 10^10^	−2.45 × 10^10^	3.87 × 10^10^
12th	8.94 × 10^9^	3.31 × 10^10^	2.02 × 10^11^	1.30 × 10^13^	2.64 × 10^13^	8.21 × 10^12^	4.39 × 10^10^	−1.76 × 10^10^	3.31 × 10^10^
11th	9.12 × 10^9^	3.55 × 10^10^	2.08 × 10^11^	1.55 × 10^13^	2.56 × 10^13^	7.41 × 10^12^	3.87 × 10^10^	−2.59 × 10^10^	2.63 × 10^10^
10th	1.04 × 10^10^	3.35 × 10^10^	1.38 × 10^11^	1.50 × 10^13^	2.27 × 10^13^	6.51 × 10^12^	4.39 × 10^10^	−2.32 × 10^10^	4.26 × 10^10^
9th	1.11 × 10^10^	2.88 × 10^10^	1.82 × 10^11^	1.53 × 10^13^	3.00 × 10^13^	9.32 × 10^12^	4.39 × 10^10^	−2.65 × 10^10^	3.66 × 10^10^
8th	1.79 × 10^10^	3.94 × 10^10^	2.60 × 10^11^	1.56 × 10^13^	4.06 × 10^13^	1.00 × 10^13^	7.31 × 10^10^	−3.32 × 10^10^	4.30 × 10^10^
7th	8.94 × 10^9^	2.05 × 10^10^	2.46 × 10^11^	1.55 × 10^13^	2.11 × 10^13^	9.52 × 10^12^	5.04 × 10^10^	−2.95 × 10^10^	3.66 × 10^10^
6th	8.94 × 10^9^	2.17 × 10^10^	2.26 × 10^11^	1.47 × 10^13^	2.15 × 10^13^	7.91 × 10^12^	6.73 × 10^10^	−3.12 × 10^10^	2.54 × 10^10^
5th	9.30 × 10^9^	2.13 × 10^10^	1.32 × 10^11^	1.53 × 10^13^	2.39 × 10^13^	7.81 × 10^12^	4.68 × 10^10^	−2.22 × 10^10^	2.37 × 10^10^
4th	9.12 × 10^9^	2.17 × 10^10^	2.47 × 10^11^	1.36 × 10^13^	2.11 × 10^13^	9.42 × 10^12^	6.87 × 10^10^	−2.22 × 10^10^	3.83 × 10^10^
3rd	1.00 × 10^10^	2.05 × 10^10^	2.57 × 10^11^	1.52 × 10^13^	2.15 × 10^13^	8.52 × 10^12^	6.58 × 10^10^	−2.52 × 10^10^	2.41 × 10^10^
2nd	9.30 × 10^9^	2.01 × 10^10^	1.45 × 10^11^	7.98 × 10^12^	2.11 × 10^13^	9.12 × 10^12^	6.65 × 10^10^	−3.35 × 10^10^	2.88 × 10^10^
Initial (1st)	6.01 × 10^9^	1.61 × 10^10^	1.40 × 10^11^	9.91 × 10^12^	2.38 × 10^13^	5.00 × 10^12^	6.16 × 10^10^	−2.29 × 10^10^	−2.67 × 10^10^
1st	3.00 × 10^9^	8.08 × 10^9^	7.86 × 10^10^	4.96 × 10^12^	1.19 × 10^13^	4.35 × 10^12^	6.03 × 10^10^	−2.24 × 10^10^	−1.84 × 10^10^
Initial (Ground)	2.28 × 10^9^	9.09 × 10^8^	5.48 × 10^10^	4.66 × 10^12^	4.87 × 10^13^	2.25 × 10^11^	3.52 × 10^9^	−8.82 × 10^9^	1.26 × 10^10^
Ground	1.14 × 10^9^	4.54 × 10^8^	2.85 × 10^10^	2.33 × 10^12^	2.43 × 10^13^	1.60 × 10^11^	3.52 × 10^9^	−8.82 × 10^9^	8.98 × 10^10^

**Table 7:** Comparison between lumped-mass model modal frequency predictions with those determined using the Green Building accelerometer data.

FE model	Modal Frequencies (Hz)					
	1st EW	1st NS	1st Torsion	2nd EW	2nd NS	2nd Torsion
Initial SLMM	1.04	1.22	1.80	3.16	4.04	7.12
Average frequency (Table 2)	0.68	0.74	1.47	2.51	2.81	5.05
Maine earthquake (Table 2)	0.66	0.74	1.45	2.39	2.81	5.05
Updated SLMM	0.66	0.76	1.45	2.39	2.81	5.05
